# A Case of Peripheral Ulcerative Keratitis Associated with Autoimmune Hepatitis

**DOI:** 10.1155/2017/3939413

**Published:** 2017-10-09

**Authors:** Hamoon Eshraghi, Aria Mahtabfar, Mohammad H. Dastjerdi

**Affiliations:** ^1^Rutgers Robert Wood Johnson Medical School, Piscataway, NJ, USA; ^2^Department of Ophthalmology, Rutgers New Jersey Medical School, Newark, NJ, USA

## Abstract

**Purpose:**

To describe a case of peripheral ulcerative keratitis in the setting of autoimmune hepatitis and possible overlap syndrome with primary sclerosing cholangitis.

**Case Report:**

A 48-year-old African American female with autoimmune hepatitis with possible overlap syndrome with primary sclerosing cholangitis presented with tearing, irritation, and injection of the left eye that was determined to be peripheral ulcerative keratitis. The patient was treated with topical and systemic steroids, immunosuppressant drugs (azathioprine and mycophenolate mofetil), a biologic (rituximab), and surgery (conjunctival resection), and the peripheral ulcerative keratitis epithelialized but ultimately led to corneal perforation.

**Conclusion:**

In this unique case, a patient with peripheral ulcerative keratitis who underwent treatment ultimately had a corneal perforation. This case may suggest a possible relationship between autoimmune hepatitis and peripheral ulcerative keratitis.

## 1. Background

Peripheral ulcerative keratitis (PUK) is an autoimmune condition believed to involve complement system activation via immune complexes in the peripheral cornea. The end result is a localized inflammatory response, leading to the enzymatic destruction of local cellular architecture; in particular, neutrophils and macrophages release collagenase and other proteases that destroy the corneal stroma [1]. The characteristic lesion is a crescent-shaped stromal inflammation of the juxtalimbal cornea. The etiology of PUK has not been completely ascertained, but consensus has centered on an autoimmune response to a localized antigen. Given the inflammatory nature of the disease, PUK is often associated with other autoimmune conditions including rheumatoid arthritis and lupus. There have been cases of association between peripheral corneal ulcers and Mooren's ulcer and viral hepatitis [2, 3]. However, there have been no cases in the literature of a presentation of PUK in the setting of autoimmune hepatitis or primary sclerosing cholangitis (PSC).

Autoimmune hepatitis is an autoimmune disorder characterized by inflammation of the liver that is often asymptomatic or can present with nonspecific symptoms such as fatigue, jaundice, abdominal pain, and even acute liver failure. The condition is more common among females and is often associated with serological markers such as anti-nuclear antibody and anti-smooth muscle antibody. The condition often has clinical characteristics that overlap with either PSC or primary biliary cholangitis (PBC), often known as overlap syndrome. We report a case of PUK in the setting of autoimmune hepatitis with possible overlap syndrome with PSC.

## 2. Case Report

A 48-year-old African American woman with a past medical history of autoimmune hepatitis and possible overlap syndrome with primary sclerosing cholangitis status post multiple stent placements and endoscopic retrograde cholangiopancreatography (ERCP) presented with tearing, redness, and irritation of the left eye. She was diagnosed with autoimmune hepatitis for over ten years before the visual symptoms began and was on a low-dose steroid maintenance treatment for the condition. She had strictures that extended to her intrahepatics with accompanying cholangitis. Histological examination confirmed hepatitis, and laboratory values confirmed a cholestatic liver enzyme profile. She also was positive for anti-nuclear and anti-smooth muscle antibodies and negative for hepatitis B and C. Visual acuity was 20/20 in the right eye and 20/40 in the left eye, and the intraocular pressures (IOPs) were 21 and 19 mmHg, respectively. Slit lamp exam showed a crescent-shaped left peripheral corneal ulcer measuring 4.5 × 0.5 mm extended from 5 to 6:30 o'clock with about 50% thinning and no anterior chamber reaction. The adjacent sclera was also inflamed with injected and edematous conjunctiva. The remainder of the eye exam was unremarkable. The patient had previously been on 10 mg of oral prednisone for prior medical history but was started on topical prednisolone acetate 1% eye drop four times daily. Prophylactic topical antibiotic eye drops, moxifloxacin, and oral doxycycline 100 mg twice daily along with preservative artificial tears were also started.

**Figure 1:**
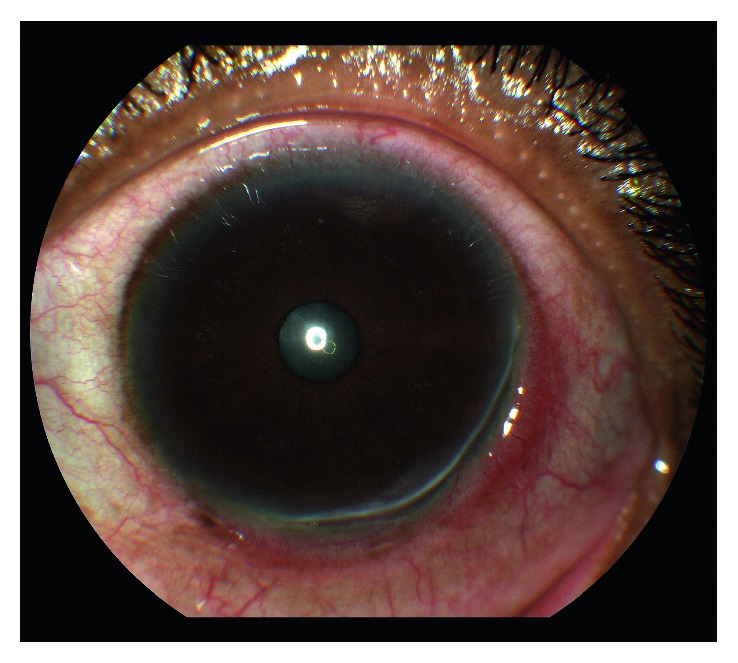
Left eye photo 6 weeks after initial presentation which shows peripheral corneal ulcer extended from 3 to 6:30 o'clock position with about 70–80% stromal thinning.

Over the next 6 weeks, there was continued worsening of the ulcer. One week after initial presentation, the patient returned with no improvement. Three weeks later, the ulcer base became widened and deepened with significant thinning of about 70%. At this time, the patient was started on azathioprine 150 mg and prednisone 60 mg daily by her rheumatologist. One month later, there was continued worsening of the ulcer extending to the 3 o'clock position with about 70–80% thinning (Figure 1()). Moreover, her right eye also showed a small area of ulceration along the limbus at the 2 o'clock position. Because of lack of progress and concern of poor medication compliance, the patient was admitted to the hospital for intravenous methylprednisolone and rituximab treatment by her rheumatologist.

While the right eye ulcer started epithelialization, the left eye showed no improvement. The patient underwent conjunctival resection in the area of ulcer in the left eye with application of fibrin glue and a bandage contact lens. Meanwhile, the patient finished a course of 1000 mg rituximab infusion along with intravenous methylprednisolone sodium succinate, 1 gram daily for 3 days, and discharged on mycophenolate mofetil and a tapering dose of oral prednisone. A month after the procedure, the ulceration in the left eye had epithelialized to about 2 or 3 clock hours, and within a subsequent month, the lesion had healed entirely with residual thinning. Visual acuities were 20/20 OD and 20/30 OS.

The patient was monitored on a monthly basis afterwards. After five months, the patient presented with 20/100 vision in the left eye. Examination showed a small linear corneal perforation near the limbus with iris prolapse at the site of previous ulcer in the left eye. The perforation was repaired with direct closure. The patient was discharged on mycophenolate mofetil. A month after the surgery, the corneal perforation had healed with superficial neovascularization, and there was a small area of iridocorneal adhesion. In a three-month follow-up visit, uncorrected visual acuity in the left eye was 20/100 that was corrected to 20/40. The intraocular pressure was measured to be 16 mmHg. There were no signs of recurrent inflammation or ulceration.

## 3. Conclusions

The autoimmune nature of PUK has led to a strong association with various autoimmune diseases. In fact, collagen vascular diseases are associated with over half of all PUK cases [4]. The dysregulated T helper cell type 1 and 2 (Th-1 and Th-2) pathways in PUK are parallel to those in other autoimmune diseases. Specifically, the reported upregulation of T-box expressed in T cells (T-bet) and self-antigen recognition in autoimmune hepatitis create a plausible etiology leading to PUK [5]. Immune complex deposition is also considered a mechanism leading to activation of the complement cascade. Antibodies associated with autoimmune hepatitis, including anti-nuclear antibody, anti-smooth muscle antibody, and peripheral anti-neutrophil cytoplasmic antibody, are involved in immune complex deposition, which may lead to activation of the complement cascade and/or promotion of T helper response. In other autoimmune diseases, such as systemic lupus erythematosus and rheumatoid arthritis, documented associations have been established with PUK, albeit with a poor understanding of the underlying mechanism of the disease [6]. There is a clear crossover between the etiology of autoimmunity involved in autoimmune hepatitis and PUK. Although various case reports associate Mooren's ulcer with hepatitis C viral infection [3], corneal complications have rarely been reported in autoimmune hepatitis. In our search of the literature, there were no prior presentations of PUK in the setting of autoimmune hepatitis, despite having similar pathophysiology.

Because of the often-asymptomatic presentation of autoimmune hepatitis, and diagnosis necessitating liver biopsy, an underreporting phenomenon may be present [7]. The current case is the first example of peripheral ulcerative keratitis associated with autoimmune hepatitis. If later studies establish a stronger association, future cases may necessitate an examination for autoimmune hepatitis in patients who present with PUK.

## Abbreviations

PUK: Peripheral ulcerative keratitis
PSC: Primary sclerosing cholangitis
PBC: Primary biliary cholangitis
ERCP: Endoscopic retrograde cholangiopancreatography
IOP: Intraocular pressure
OD: Right eye
OS: Left eye
Th-1: T helper cell type 1
Th-2: T helper cell type 2
T-bet: T-box expressed in T cells.
